# Impact of pharmacy intervention on influenza vaccination acceptance: a systematic literature review and meta-analysis

**DOI:** 10.1007/s11096-021-01250-1

**Published:** 2021-05-28

**Authors:** Erin Murray, Karolina Bieniek, Michael del Aguila, Sonya Egodage, Severine Litzinger, Assia Mazouz, Henry Mills, Jan Liska

**Affiliations:** 1 Doctor Evidence, Santa Monica, CA USA; 2 Sanofi Pasteur, Lyon, France; 3 Sanofi General Medicines, Trade and Revenue Management, Paris, France; 4 Global Vaccines Medical, Sanofi Pasteur, Lyon, France; 5 Sanofi General Medicines, Paris, France, 54 rue La Boétie, Sanofi, Paris, France

**Keywords:** Acceptance, Influenza, Pharmacist, Vaccine

## Abstract

*Background* Vaccination plays an important role in the prevention of influenza. Channels that improve vaccination adherence can play a vital part in improving patient care. This study seeks to inform the design and implementation of pharmacy interventions at scale on improving influenza vaccination rates. *Aim of the review* The aim of this study was to identify key success factors for effective pharmacy intervention design and implementation to improve vaccination acceptance rates in influenza. *Methods* A systematic search of MEDLINE, Embase, and Cochrane CENTRAL was performed to find literature on influenza vaccinations delivered at pharmacies, pharmacist-delivered influenza vaccinations, or influenza vaccination campaigns originating in the pharmacy setting. A meta-analysis using a random effects model estimated the impact of pharmacy intervention on vaccination rates (assessed as relative risk [RR] and 95% confidence intervals [95% CI]). *Results* A total of 1221 studies were found that met the search criteria, of which 12 were selected for the literature review following eligibility screening. A meta-analysis of studies that contained binary total population and vaccination rate data was conducted on 6 studies, including 3182 participants, the vaccination rate was 24% higher in those who used the pharmacy-based intervention compared with those who used standard care [RR (95% CI) 1.24 (1.05, 1.47)]. Two separate sensitivity analyses were run for the vaccination rate. In participants aged ≥ 65 years, the vaccination rate was 3% higher in those who received the pharmacy-based intervention compared with those who received standard care; however, this change was not significant [RR (95% CI) 1.03 (0.86, 1.24)]. Additionally, a qualitative review showed that more successful pharmacy-based interventions were those with the more active involvement of pharmacists in routine care. This included regular checkup of vaccine status, proactive conversations and recommendations about vaccination, and pharmacy-based immunization programs, with specific vaccination days. In-pharmacy communication rather than passive information, such as through leaflets and posters was also more effective. *Conclusion* Pharmacists can play a significant role to improve patient treatment, adherence, and outcomes associated with influenza vaccines. Once pharmacy-based immunization is established, proactive involvement of is key to ensure successful program implementation and results. Expanding access for pharmacists and pharmacy intervention to provide vaccinations may increase vaccination acceptance and could be a valuable intervention in patient care. Additional studies should consider high-risk populations to inform optimal design and implementation strategies.

**Supplementary Information:**

The online version contains supplementary material available at 10.1007/s11096-021-01250-1.

## Impacts on practice

- This review shows the positive impact of expanding the pharmacy role in immunization protocols.
- Given the current state of clinical affairs and vaccination efforts worldwide, providing novel information relevant to understanding the influence of pharmacists and pharmacy intervention on vaccination acceptance is incredibly pertinent and necessary.
- This review also shows that there seems to be a greater impact when an active and clearly defined pharmacy intervention is used compared to passive interventions.

## Background

Influenza-associated respiratory illness presents a sizable global disease burden and is responsible for an estimated 291,243–645,832 (4.0–8.8 per 100,000 individuals) deaths annually among all ages [1]. The influenza-associated mortality rate is highest among adults ≥ 75 years (51.3 to 99.4 per 100,000 individuals), and the highest range of deaths among all ages is in sub-Saharan Africa (27,813–163,074; 17%) [2]. It has been estimated that between about 70% and 90% of seasonal flu-related deaths have occurred in people 65 years and older [3].

However, influenza vaccination provides a valuable tool for combating the influenza burden. During 2016–2017, the United States Centers for Disease Control (CDC) reported that the flu vaccination prevented an estimated 5.3 million influenza-related illnesses, 2.6 million influenza-associated medical visits, and 85,000 influenza-associated hospitalizations in the US. In seasons when the vaccine viruses matched circulating strains, the vaccine has been shown to reduce the risk of physician visits for the flu by 40–60%. In recent years, flu vaccines have reduced the risk of flu-associated hospitalizations among adults on average by about 40%. A 2018 study showed that from 2012 to 2015, flu vaccination among adults reduced the risk of being admitted to an intensive care unit (ICU) with flu by 82% [4].

Despite the body of evidence on the effectiveness of influenza vaccines in preventing morbidity and mortality, barriers to vaccination still remain for some patients [5]. The CDC reported that vaccination rates in the US reached only 37% during the 2017–2018 season, down 6.2% from the previous season [6]. In Europe, most countries are still well below the recommended 75% coverage rate for older adults, ranging from 2% to 72.8%, with the large variation attributable to differences in government policy and healthcare delivery systems [7].

Barriers to achieving recommended vaccination rates include a lack of interventions that increase patient demand, a lack of access to a regular source of care, and missed opportunities for physicians to collaborate with alternative healthcare providers to offer preventative healthcare recommendations. One option to address these barriers is to leverage pharmacy-based delivery of vaccinations. For patients, pharmacists and pharmacy-based care offer a convenient and accessible alternative for immunization services. Pharmacists are viewed as trusted health professionals and are easily available to the public in rural areas and other areas with few healthcare professionals [8]. Additionally, expanding access through the use of non-traditional settings such as pharmacies may combat the consistent low coverage rates by improving vaccination uptake and reaching people in settings other than traditional physicians’ offices [9]. This systematic literature review (SLR) and meta-analysis was performed to explore the impact of pharmacist and pharmacy channel on influenza vaccination acceptance and uptake.

## Aim of the review

The aim of this study was to explore the mechanism of impact for effective pharmacy intervention design and implementation at scale, to improve vaccination acceptance rates in influenza.

## Methods

### Study selection

A search of MEDLINE, Embase, and Cochrane CENTRAL was performed to find English language literature published from inception to February 22, 2018, on influenza vaccinations delivered at pharmacies, pharmacist-delivered influenza vaccinations, or influenza vaccination campaigns originating in the pharmacy setting. Manual searches of relevant conference proceedings and review of reference lists from similar reviews were crawled for potential additional studies. The search strategy was conducted by a medical librarian (MC) and reviewed by a clinical methodologist (EM). Detailed search strategies can be found in the Supplement. The methods were adapted from standard guidelines provided by the Cochrane Handbook for Systematic Reviews of Interventions [10]. Results were reported according to the Preferred Reporting Items for Systematic Reviews and Meta-Analyses (PRISMA) guidelines [11].

Study eligibility was guided by the population, intervention, comparator, outcome (PICO) framework as described in the Cochrane Handbook [10]. Included studies were randomized and non-randomized controlled trials or observational studies of any patient population. Studies were required to include a pharmacy intervention, defined as: (1) pharmacist-delivered influenza vaccine, (2) pharmacist-delivered influenza vaccination campaigns, or (3) influenza vaccination campaigns originating in the pharmacy setting compared to any other intervention. Single-arm studies were excluded. Outcomes of interest included: (1) vaccination rate and (2) characteristics of successful programs. The pre-defined PICO criteria for the studies that were included in this review are outlined in the Supplement.

Literature was identified by evaluating study eligibility against the PICO framework. All studies were initially screened at the title and abstract level by a single reviewer, then by a second reviewer for quality control (MS, HT) in which a subset of studies were assessed and an agreement score reached. Subsequent full-text screening was conducted on potentially relevant references identified at title/abstract screening, after removal of duplicate publications. Full-text screening was conducted by two blinded medical librarians (HT, MS). Any discrepancies were resolved via discussion. Reviewers recorded specific reasons for study exclusion during both stages of screening.

### Data extraction

Study extraction was conducted by a clinical research analyst and reviewed for quality control by two independent, blinded reviewers. The following data were extracted from each study: (1) study design and characteristics, (2) patient baseline characteristics including demographics and inclusion/exclusion criteria, (3) outcomes of interest as described in the protocol. Data was extracted using the DOC Extract 2.0 platform (Doctor Evidence: DOC Data, Version 2.0 Santa Monica, CA). Included studies were assessed for risk of bias using the Cochrane Collaboration tool for assessing risk of bias in randomized trials [10] and The Newcastle-Ottowa Scale (NOS) for assessing quality of non-randomized studies in meta-analyses [12].

### Statistical methods

All analyses were conducted using the DOC Data 2.0 advanced web-based platform (Doctor Evidence: DOC Data, Version 2.0 Santa Monica, CA). The R “metafor” software package was used to perform the meta-analysis. Analysis of heterogeneity (ANOHE) assessed the appropriateness of the included studies for each analysis. The Q-test for heterogeneity is reported for each outcome. The risk ratio (RR) was analyzed for vaccination rates with corresponding 95% confidence interval (CI). Random effects models using the DerSimonian and Laird (DL) estimator were used during the analysis. Since a random-effects DerSimonian and Laird model was run, the RR of each study was pooled together and weighted by the inverse variance or each estimate allowing for slight variation in different studies variance estimates.

Three separate analyses were run for vaccination rate outcomes. The main analysis included studies that contained binary total population and vaccination rate data. Two sensitivity analyses were run in order to determine whether significant differences would be identified in the vaccination rate when different outcome assessment types were included in analysis. A primary sensitivity analysis was run on seven studies and included studies from the main analysis as well as one study reporting only relative risk data. The second sensitivity analysis included the studies from the primary sensitivity analysis as well as three additional studies that reported on data as “pre- “ and “post-” pharmacist/pharmacy involvement law change. This sensitivity analysis was performed to determine if the results changed when the comparator was not a concurrent control group but rather a before and after comparison. Additional subgroup analyses were done for high-risk groups including the elderly (age ≥ 65 years) cohort and those not vaccinated in a previous flu season.

## Results

**Fig. 1:**
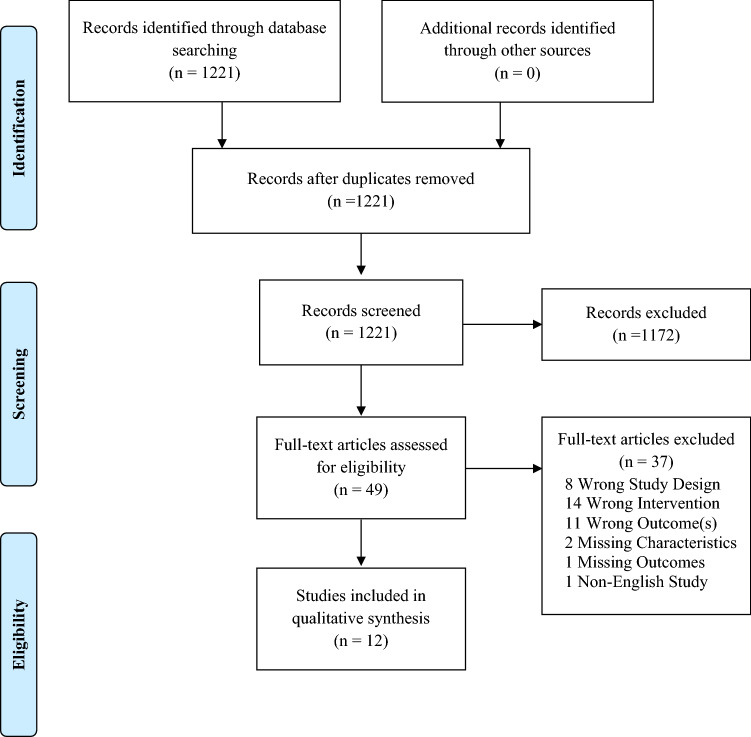
Study flow diagram (PRISMA)

A total of 1221 unique studies were found in the search, 49 studies were selected after title/abstract screening and 12 were selected after full-text screening (Fig. 1). Eleven studies were journal articles, the majority of which were published between 2014 and 2018; one article was a meeting abstract, published in 2017 [13–24].

All but two studies were conducted in the United States [14, 23]. All studies were from 2000 to2018, with two studies published in 2016 and two in 2017. Most studies were observational comparative, with three randomized control trials and one non-randomized controlled trial. Study populations ranged from 394,339 participants [20] to 89 participants [22] (Table 1).

**Table. 1:** Characteristics of included studies

First Author	Year	Title	Design	Study N	Age	Female (%)
Edwards HD [13]	2012	A pharmacist visit improves diabetes standards in a patient-centered medical home (PCMH)	Non-Randomized Controlled Trial	323	NR	199 (61.6)
Ginson S.H. [14]	2000	Impact on vaccination rates of a pharmacist-initiated influenza and pneumococcal vaccination program	Randomized Controlled Trial	102	NR	68 (66.7)
Grabenstein JD [15]	2001	Effect of vaccination by community pharmacists among adult prescription recipients	Retrospective Cohort Study	4403	64.8 (SD ± 15.2)	1212 (58)
Hill JD [16]	2017	Development of a Pharmacy Technician-Driven Program to Improve Vaccination Rates at an Academic Medical Center	Controlled Before and After Trial	142	NR	NR
Isenor JE [17]	2016	Impact of pharmacists as immunizers on influenza vaccination coverage in the community-setting in Nova Scotia, Canada: 2013–2015	Retrospective Cohort Study	NR	NR	NR
Klassing HM [18]	2018	Evaluation of Pharmacist-Initiated Interventions on Vaccination Rates in Patients with Asthma or COPD	Randomized Controlled Trial	831	NR	NR
Loughlin SM [19]	2007	Pharmacist-managed vaccination program increased influenza vaccination rates in cardiovascular patients enrolled in a secondary prevention lipid clinic	Retrospective Cohort Study	742	NR	173 (23.3)
Mohammad I. [20]	2017	Outcomes of chronic care management (CCM) in primary care practice	Non-Randomized Controlled Trial	89	NR	NR
Padiyara RS [21]	2011	Clinical pharmacist intervention and the proportion of diabetes patients attaining prevention objectives in a multispecialty medical group	Retrospective Cohort Study	642	NR	342 (53.3)
Robison SG [22]	2016	Impact of pharmacists providing immunizations on adolescent influenza immunization	Retrospective Cohort Study	394339	11 – 17	NR
Usami T [23]	2009	Impact of community pharmacists advocating immunization on influenza vaccination rates among the elderly	Cluster RCT	1867	NR	1271 (68.1)
Wang J [24]	2014	Racial and ethnic disparities in influenza vaccinations among community pharmacy patients and non-community pharmacy respondents	Retrospective Cohort Study	8922	NR	4932 (55.3)

Risk of bias available in supplement

A summary of the risk of bias assessment for all the selected studies is available in Supplement Table 3. Risk of bias based on the Cochrane assessment tool for RCTs was judged as high for all three studies in blinding for both personnel and outcome assessment and high for other sources of bias in two out of the three studies. Other criteria were judged as low or unclear risk. The results of the Newcastle Ottawa Scale assessment tool for observational studies indicated possible bias associated with the representative of the study cohorts, but most studies did select a control group from the same population as the exposed group. Most studies also controlled for effect modifiers including demographics (age, gender, etc.) and one controlled for social status (poverty level, education, etc.).

**Fig. 2:**
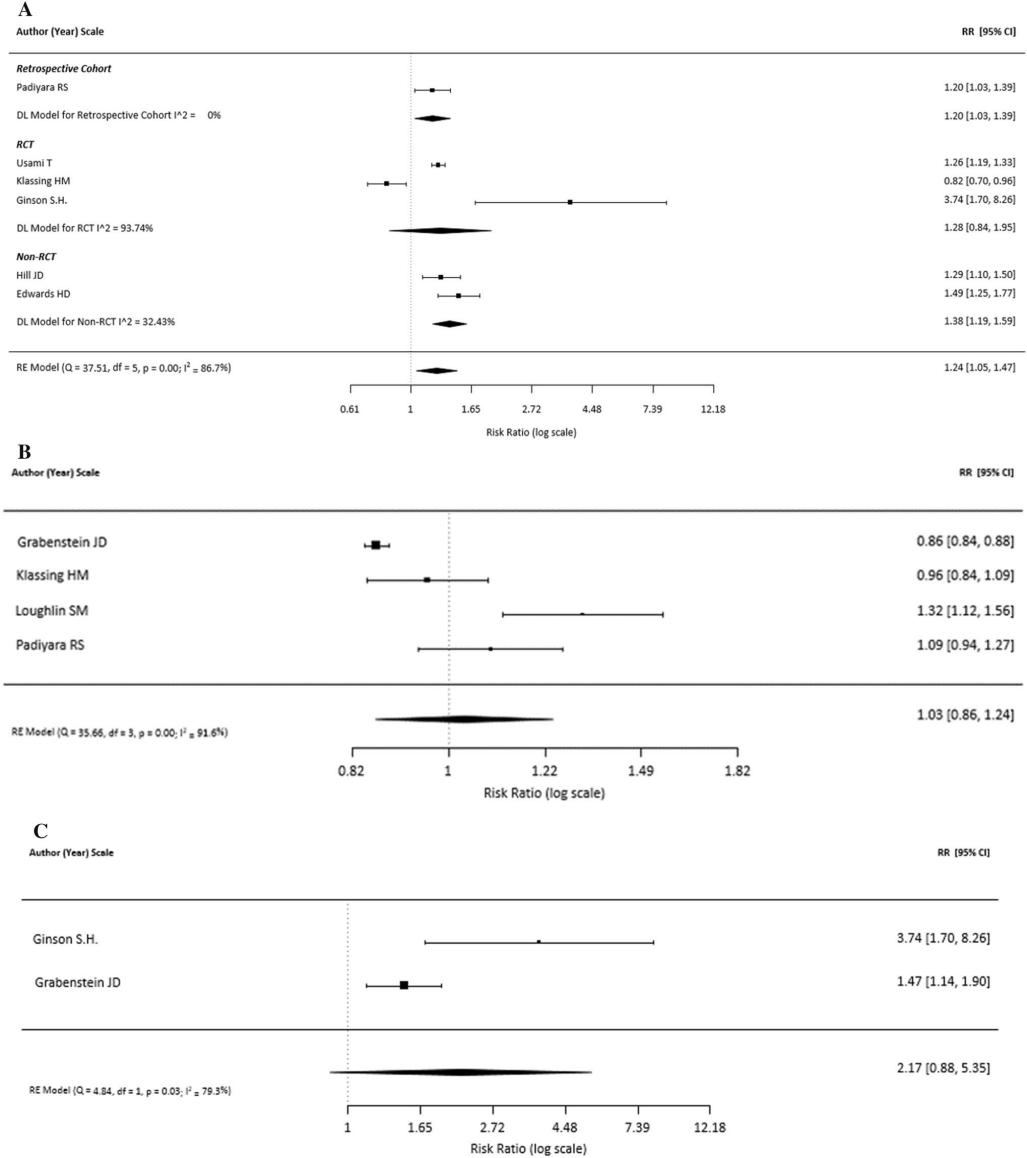
Base case and Subgroup Meta-Analysis of Vaccination Rates Results favoring pharmacist intervention vs standard care are to the right of the figure **a** Base-case analysis of vaccination rate by study design Klassing 2018 study was specific to a cohort of participants with asthma and/or COPD, and therefore may have had a more rigorous standard of care regarding vaccinations leading to less influence of pharmacist intervention. **b** ≥ 65 year subgroup analysis of vaccination rates **c** No prior history of ion subgroup analysis of vaccination rates

Six studies were included in the main meta-analysis for vaccination rate (Fig. 2). These studies reported on total population, number vaccinated in those using pharmacy intervention and number vaccinated in those using standard care and therefore were included in the main analysis [13, 14, 16, 18, 21, 23]. The results (6 studies, 3182 participants) show that vaccination was 24% more likely in those who used the pharmacy intervention compared with those who used standard care [RR (95% CI): 1.24 (1.05, 1.47)]. However, the overall analysis had high heterogenicity (I^2^ = 86.7%).

### Sensitivity analysis

Seven studies were included in a primary sensitivity analysis (see Supplement Fig. 1). The results show that vaccination was 22% more likely in those who used the pharmacy intervention compared with those who used standard care. Similar to the base case results, the overall analysis had high heterogenicity (I^2^ = 84.2%).

A second sensitivity analysis (see Supplement Fig. 1) included ten studies. In this scenario, vaccination was 27% more likely in those who used the pharmacy intervention compared with those who used standard care [RR (95% CI): 1.27 (1.09, 1.48)]. The overall analysis had high heterogenicity (I^2^ = 97.2%).

### Subgroup analysis

A subgroup analysis of the elderly patients included four studies (2860 participants) and indicated that vaccination was 3% [RR (95% CI): 1.03 (0.86, 1.24)] more likely in those who used the pharmacy intervention compared with those who used standard care; however, this difference was not significant (Fig. 2). The overall analysis had high heterogenicity (I^2^ = 91.6%).

A subgroup analysis of participants who had not received the influenza vaccination in the previous flu year included two studies (660 participants) and indicated that vaccination was 117% [RR (95% CI): 2.17 (0.88, 5.35)] more likely in those who used the pharmacy intervention compared with those who used standard care, however, this difference was not significant (Fig. 2). The overall analysis had high heterogenicity (I^2^ = 79.3%).

### Qualitative review

The qualitative review sought to identify key factors that contributed to more successful pharmacy intervention. More successful interventions employed an active rather than passive pharmacy role. The interventions with explicit protocols involving pharmacists and pharmacy intervention in routine care, such as electronic medical records (EMR) review, patient history and physical, and medication management improved vaccination rates over standard care or passive information through leaflets and posters (Table 2).

**Table. 2:** Included Studies Vaccination Rates

First Author	Location	Intervention	Comparator	Intervention N	Comparator N	Intervention Vaccinated #	Comparator Vaccinated #	Risk Ratio (95% CI)
Edwards HD [13]	United States	Seen by pharmacist EMR Reviewed Pharmacist history, physical exam and lab testing	Seen by physician	113	210	84	105	1.49 (1.25, 1.77)
Ginson SH [14]	Canada	Pharmacist- patient vaccination education Vaccination pamphlet Conditional order for vaccination written by pharmacist Vaccination required physician signature before administration	Standard care by physician	28	37	17	6	3.74 (1.70, 8.26)
Grabenstein JD [15]	United States	Pharmacists authorized to administer medications (Washington)	Pharmacists not explicitly authorized to administer medications, nor were any known to do so (Oregon)	4422	4384	1443	1606	0.89 (0.84, 0.94)
Hill JD [16]	United States	Intervention by pharmacist technician Phone call reminder and/or face-to-face discussion with nursing staff Immunization status and EMR review of hospital unit	Standard care by nurse	70	72	65	52	1.29 (1.10, 1.50)
Klassing HM [18]	United States	Pharmacist initiated phone call In-store advertising On-site immunizations	In-store advertising On-site immunizations	77	70	56	62	0.82 (0.70, 0.96)
Loughlin SM [19]	United States	Pharmacist-managed vaccination program Screened and offered influenza vaccination under a standing-order protocol	No formal immunization program	266	476	202	183	1.98 (1.73, 2.25)
Mohammad I [20]	United States	Pharmacist-led medication management, care coordination, and management at transitions of care	Usual care treatment	67	22	*	*	1.14 (0.90, 1.45)
Padiyara RS [21]	United States	Pharmacist-patient vaccination education Pharmacist direct drug therapy management and preventative care services Pharmacist autonomy in patient assessment and education Pharmacist review and adjust medication therapy, order labs, determine follow-up	Standard care and screenings provided by primary care physician, nurse practitioner, or physician assistant	321	321	182	152	1.20 (1.03, 1.39)
Robison SG [22]	United States	Pharmacists delivered vaccinations without prescription	Pharmacist delivered vaccinations by physician prescription only	195441	198898	51206	37194	1.40 (1.38, 1.42)
Usami T [23]	Japan	Pharmacist displayed informational poster on vaccination Physically handed informational leaflet to patient	Pharmacist did not provide poster or leaflet information for vaccinations	911	952	743	618	1.26 (1.19, 1.33)

*Only relative risk reported, numbers were back-calculated

## Discussion

To our knowledge, this is the first systematic review and analysis undertaken to identify key success factors for effective pharmacy intervention design and implementation at scale, to improve vaccination acceptance rates.

Overall, we found that pharmacy-based interventions lead to an increase in vaccination acceptance of up to 27% compared to standard of care, and up to 117% for those who have not received influenza vaccination in the previous year. Enabling pharmacists (and others within the pharmacy-care setting) to provide vaccinations can increase the probability of vaccination acceptance and is a useful tool in providing adequate patient care. Instances where protocols for vaccinations involved pharmacists participating in routine care led to higher vaccination rates over standard care or passive information. Specifically, strategies involving immediate and direct communication between the pharmacist and patient largely contributed to the increase in vaccination rates. Programs developed by pharmacists based on known determinants of vaccinations and vaccination behavior appeared to increase patient awareness. Increasing awareness allows patients to become their own advocates for chronic care, which could also contribute to improved outcomes [13, 14].

An insignificant difference was found in the subgroup analysis of elderly patients. This insignificant difference is likely related to the fact that most countries that have vaccination guidelines also include specific recommendations for older adults [25]. Considering these higher regulations and priorities placed on the elderly, along with the higher proportion vaccinated in this group, the impact of pharmacy-based care as an alternative to standard care was less than what was seen in the general population. A review on the impact of pharmacists as immunizers also compared vaccine administered by pharmacists versus provisions by traditional providers with no pharmacy involvement [26]. Those authors also found that pharmacist involvement in immunization resulted in increased uptake of immunizations. They also noted that there was an established positive impact of pharmacists as immunizers regardless of the role (educator, facilitator, administrator) or type of vaccine administered (e.g. influenza, pneumococcal) [26].

Some strengths of our study include the use of rigorous systematic research methods to conduct the search and analysis of relevant information. The use of a control arm to compare against pharmacist and pharmacy interventions provided a baseline from which we were able to ascertain the impact of such interventions. Additionally, sensitivity and sub-group analysis provided additional validation of the results.

Limitations of this analysis include the following. The consideration of pharmacy intervention compared with standard of care limited the number of studies found in this review compared to all research on overall vaccination rates and location. A majority of the analysis was based on data from observational studies, which have an inherent source of bias but do collect information on behavior in the real-world setting. Indeed, the risk of bias assessments conducted on both RCTs and observational studies indicated many sources of bias present in the studies which may have affected the results. All analyses had high heterogenicity ranging from 79.3 to 97.2%, which indicates strong variation in the results and is likely a result of the difference in study designs, pharmacy interventions, and standard of care used in each study. Pharmacy interventions ranged from passive distribution of leaflets to protocols requiring active pharmacist role in vaccinations of patients, including regular checkup of vaccine status, proactive recommendations and conversation about vaccination, pharmacy-based immunization programs, and set of specific vaccination days in pharmacy. Standard of care was author-defined and may have varied greatly between studies—no formal immunization program, in-store advertising, standard care and screening by physicians or nurses are some examples. Additionally, some studies focused on high-risk cohorts, which may have higher regulations in place for vaccinations, which could skew the results. Finally, most studies took place in the United States, limiting the generalizability of these results. The differences in the study initiatives might be partly attributed to the differences in political leadership and healthcare system organizations around the globe. In the United States, pharmacists may not be as involved in routine health care and there exists no political leadership promoting their involvement; while in many parts of the world, pharmacists are the first-line health care practitioners who are already consulted regularly in current practice. However, the lack of studies from other regions may still reflect a gap in the research around pharmacy-based vaccination initiatives and calls for additional studies for the impact of pharmacists in different countries.

As the first published meta-analysis of the impact of pharmacists and pharmacy-based intervention on influenza vaccination rates, this review provides novel information relevant to understanding the influence of pharmacists on vaccination acceptance. This review also shows that there seems to be a greater impact when an active and clearly defined pharmacist and pharmacy intervention is used compared to passive interventions. These factors could contribute to further research on improving pharmacy-based vaccination initiatives. These preliminary results would benefit from future confirmation, updated search results, even across different vaccine types, or further exploration based on a larger number of pharmacy interventions with results from different countries. Additional evaluation of administration of the influenza vaccine by pharmacists may be an important area of future research, including pharmacy-technicians as the need for those who administer immunizations, including screening and medical recommendation services increases.

## Conclusion

This review supports the positive impact of expanding the pharmacist’s and pharmacy-based roles in immunization protocols. The results suggest that pharmacy centered interventions remain a promising tool to improve vaccination acceptance rates. The recent COVID-19 pandemic has affected healthcare delivery, and has shown the need for pharmacy in proximity to primary care, even beyond influenza.

## Supplementary Information

Supplementary file 1 (DOCX 118 KB)

## Data Availability

The datasets generated during and/or analysed during the current study are already publicly available as independently published studies. Further information on the analysis may be made available from the corresponding author on reasonable request.
