# Supplementary material for: Impact of pharmacy intervention on influenza vaccination acceptance: a systematic literature review and meta-analysis
Source: Int J Clin Pharm. 2021 May 28;43(5):1163–72. doi: 10.1007/s11096-021-01250-1 (PMC8161720; doi:10.1007/s11096-021-01250-1)
Supplement: Supplementary file 1 — (DOCX 118 KB) [file 11096_2021_1250_MOESM1_ESM.docx]

Impact of Pharmacy Intervention on Influenza Vaccination Acceptance: A Systematic Literature Review and Meta-Analysis

Erin Murray, MPH;^1^ Karolina Bieniek, MBT;^2^ Michael del Aguila, PhD; ^1^ Sonya Egodage, BS; ^1^  Severine Litzinger, MBA;^3^ Assia Mazouz, MS;^4^ Henry Mills,MSc, MSB;^5^ Jan Liska, MS^6^

# SUPPLEMENT

- Table 1: Search strategies (PubMed and Embase)
- Table 2: PICO Table
- Figure 1: Sensitivity Analyses – Forest Plots
- Table 3: Risk of Bias

Table 1: Search Strategies

|  | DATABASE: *PubMed* |  |
| --- | --- | --- |
|  | **DATE SEARCHED: 3/19/18** |  |
|  |  |  |
| 1 | (Pharmacists[mh] OR Pharmacy[tiab] OR pharmacies[tiab] OR pharmacist*[tiab] OR chemist[tiab] OR chemists[tiab]) | 67307 |
| 2 | ("Vaccines"[Mesh] OR "Vaccination"[Mesh] OR vaccine*[tiab] OR vaccination*[tiab] OR immunization*[tiab] OR immunisation*[tiab] OR immunized[tiab] OR immunised[tiab] OR inoculat*[tiab] OR booster*[tiab] OR Biothrax[tiab] OR Vaxchora [tiab] OR Pentacle[tiab] OR ActHIB[tiab] OR Hiberix[tiab] OR PedvaxHIB[tiab] OR Trumenba[tiab] OR MenB[tiab] OR Bexsero[tiab] OR Menveo[tiab] OR MCV4[tiab] OR Menactra[tiab] OR MenACWY[tiab] OR Boostrix[tiab] OR Adacel [tiab] OR Quadrucel[tiab] OR Kinrix[tiab] OR Infanrix[tiab] OR Daptacel [tiab] OR Pneumovax 23[tiab] OR PPSV23[tiab] OR Prevnar 13[tiab] OR PCV13[tiab] OR Typhim VI[tiab] OR Vivotif[tiab] OR ProQuad[tiab] OR MMR[tiab] OR MMrV[tiab] OR M-M-R II[tiab] OR Ipol[tiab] OR IPV[tiab] OR Zostavax[tiab] OR Shingrix[tiab] OR Afluria[tiab] OR Fluad[tiab] OR Fluarix[tiab] OR Flublok[tiab] OR Flucelvax[tiab] OR Flulaval[tiab] OR Flumist[tiab] OR Fluvirin[tiab] OR Fluzone [tiab] OR Ixiaro[tiab] OR Gardasil[tiab] OR Imovax[tiab] OR RabAvert [tiab] OR Rotarix[tiab] OR rotateq [tiab] OR ACAM2000[tiab] OR Twinrix[tiab] OR Havrix[tiab] OR vaqta[tiab] OR Engerix-B[tiab] OR Recombivax HB[tiab] OR Varivax [tiab] OR YF-vax[tiab]) | 488289 |
| 3 | 1 AND 2 | 958 |
| 4 | 3 NOT (animals[mh] NOT humans[mh]) | 949 |
| 5 | 4 AND eng[la] | 873 |

|  | EMBASE SEARCH |  |
| --- | --- | --- |
|  | **DATE SEARCHED: 2/22/18** |  |
|  | **EMBASE SEGMENT USED: 1974 - 2018 Feb 21** |  |
|  |  |  |
| # | Searches | Results |
| 1 | *pharmacist/ or (pharmacy or pharmacies or pharmacist$ or chemist or chemists).ti,ab. | 129792 |
| 2 | exp *immunization/ or exp *vaccine/ or (vaccine* or vaccination* or immunization* or immunisation* or immunized or immunised or inoculat* or booster* or Biothrax or Vaxchora or Pentacle or ActHIB or Hiberix or PedvaxHIB or Trumenba or MenB or Bexsero or Menveo or MCV4 or Menactra or MenACWY or Boostrix or Adacel or Quadrucel or Kinrix or Infanrix or Daptacel or Pneumovax 23 or PPSV23 or Prevnar 13 or PCV13 or Typhim VI or Vivotif or ProQuad or MMR or MMrV or M-M-R II or Ipol or IPV or Zostavax or Shingrix or Afluria or Fluad or Fluarix or Flublok or Flucelvax or Flulaval or Flumist or Fluvirin or Fluzone or Ixiaro or Gardasil or Imovax or RabAvert or Rotarix or rotateq or ACAM2000 or Twinrix or Havrix or vaqta or Engerix-B or Recombivax HB or Varivax or YF-vax).ti,ab. | 544546 |
| 3 | 1 and 2 | 1989 |
| 4 | 3 not ((exp animal/ or nonhuman/) not exp human/) | 1895 |
| 5 | limit 4 to english language | 1766 |
| 6 | limit 5 to (article or article in press or conference paper) | 768 |
| 7 | limit 5 to (yr="2016 -Current" and (conference abstract or "conference review")) | 165 |
| 8 | 6 or 7 | 933 |
| 9 | remove duplicates from 8 | 916 |

| The Cochrane Library (Wiley) | |  |
| --- | --- | --- |
| Date of search: 2/22/18 | |  |
|  |  |  |
| Cochrane Central Register of Controlled Trials (Clinical Trials), Issue 2, 2018 | | Results in CENTRAL: 5 |
| Cochrane Database of Systematic Reviews (Cochrane Reviews), Issue 3, 2018 | | Results in Cochrane Reviews: 3 |
| Database of Abstracts of Reviews of Effects (Other Reviews), Issue X, 2015 | | Results in Other Reviews: X |
| Health Technology Assessment Database (Technology Assessments), Issue X, 2015 | | Results in Technology Assessments: X |
|  |  |  |
|  |  |  |
| # | Searches | Results |
| #1 | [mh Pharmacists] or (pharmacy or pharmacist* or chemist or chemists):ti,ab,kw | 4558 |
| #2 | [mh vaccines] or [mh vaccination] or (vaccine* or vaccination* or immunization* or immunisation* or immunized or immunised or inoculat* or booster* or Biothrax or Vaxchora or Pentacle or ActHIB or Hiberix or PedvaxHIB or Trumenba or MenB or Bexsero or Menveo or MCV4 or Menactra or MenACWY or Boostrix or Adacel or Quadrucel or Kinrix or Infanrix or Daptacel or Pneumovax 23 or PPSV23 or Prevnar 13 or PCV13 or Typhim VI or Vivotif or ProQuad or MMR or MMrV or M-M-R or Ipol or IPV or Zostavax or Shingrix or Afluria or Fluad or Fluarix or Flublok or Flucelvax or Flulaval or Flumist or Fluvirin or Fluzone or Ixiaro or Gardasil or Imovax or RabAvert or Rotarix or rotateq or ACAM2000 or Twinrix or Havrix or vaqta or Engerix-B or Recombivax HB or Varivax or YF-vax):ti,ab,kw | 19714 |
| #3 | #1 and #2 | 71 |
| #4 | #3 not (pubmed or embase):an in Cochrane Reviews (Reviews and Protocols) and Trials | 8 |

Table 2: PICO Table

| PICO | Inclusion Criteria | Exclusion Criteria |
| --- | --- | --- |
| Population | - Any population (healthy or high risk) | - N/A |
|  | Sub-groups or special populations considered separately if data are available:   - Age 50 -65 years - Age >=65 years - Vaccination Status   - Previously vaccinated   - Not routinely vaccinated   - Never vaccinated - People living with Chronic Conditions - Specific region/ country |  |
| Intervention | - Pharmacist Delivered Vaccinations (Influenza Vaccine) - Pharmacist Delivered Vaccination Campaigns (Influenza Vaccine)   - Face to Face   - Digital/Web-based/Telephone - Vaccination Campaigns Originating in the Pharmacy Setting (Influenza Vaccine) | - N/A |
| Comparator | - Any (GP office/ vaccination centers/ surgeries) | - N/A |
| Outcome | - Vaccination Rate - Characteristics of successful programs |  |
| Timing | - Any | - N/A |
| Setting | - Any | - N/A |
| Study Design | **Include:**   - - Randomized Control Trials   - Systematic reviews/meta-analyses   - Non-randomized clinical trials   - Observational studies, comparative | - - Observational, non-comparative   - Pre-clinical   - Pooled analyses   - Case reports/series   - Prognostic course/factor studies   - Modeling studies - Narrative reviews |
| Language | - English | - Non-English |
| Publication date restriction | - Databases: Inception - present - Conferences: Last 2 years |  |

Figure 1: Sensitivity Analysis – Forest Plots

(Results favoring pharmacist intervention vs standard care are to the right of the figure)

- Main Analysis (most conservative estimate): Studies that contained binary total population and vaccination rate data.
- Studies reporting total population and number vaccinated in those receiving vaccination after pharmacist intervention and those receiving vaccination after standard care (Edwards, Ginson, Hill, Klassing, Padiyara, Usami).
- Sensitivity Analysis 1: Studies from the main analysis as well as studies reporting only relative risk data (Figure 1A)
- Studies reporting total population and number vaccinated in those receiving vaccination after pharmacist intervention and those receiving vaccination after standard care.
- Studies reporting relative risk only (Mohammad).
- Sensitivity Analysis 2 : Studies from sensitivity analysis 1 as well as studies that reported cohort data dependent on law changes allowing pharmacists to administer vaccination. (Figure 1B)
- Studies reporting total population and number vaccinated in those receiving vaccination after pharmacist intervention and those receiving vaccination after standard care.
- Studies reporting relative risk only.
- Studies reporting data as pre- and post- pharmacist involvement law change (Grabenstein, Loughlin, Robison).

Figure 1A: Vaccination Rate - Sensitivity Analysis 1


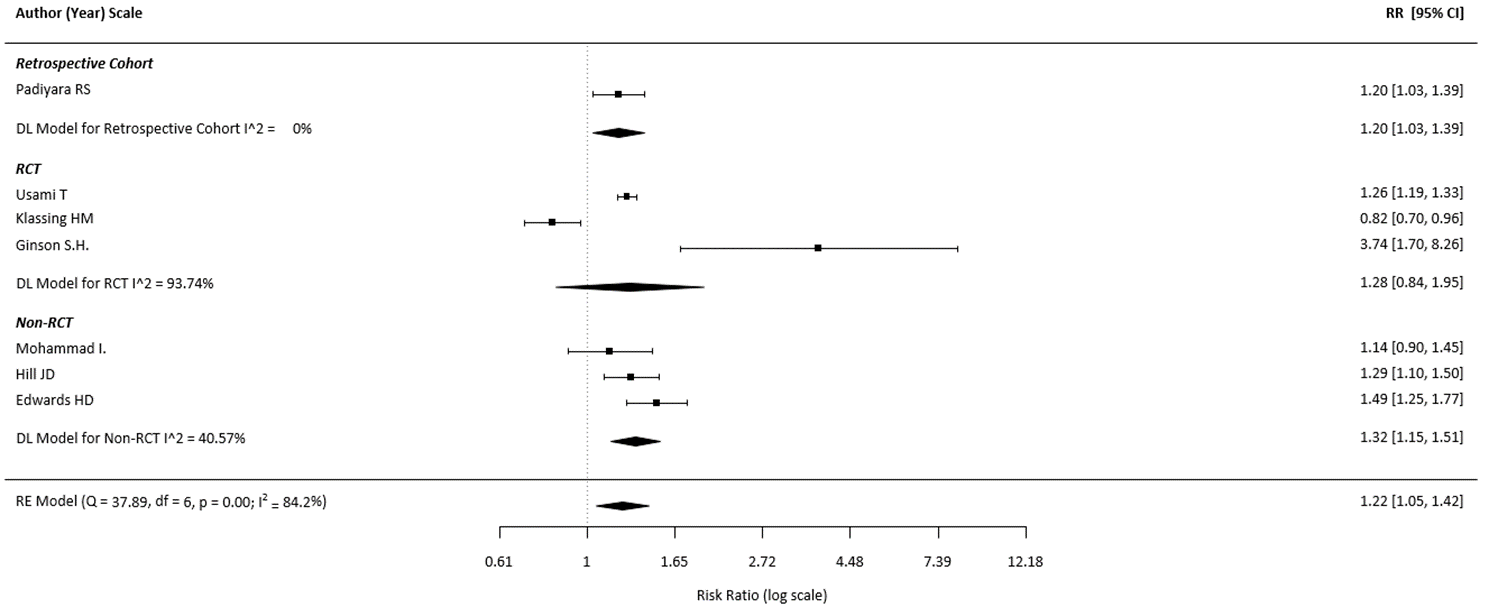


Figure 1B: Vaccination Rate -Sensitivity Analysis 2:


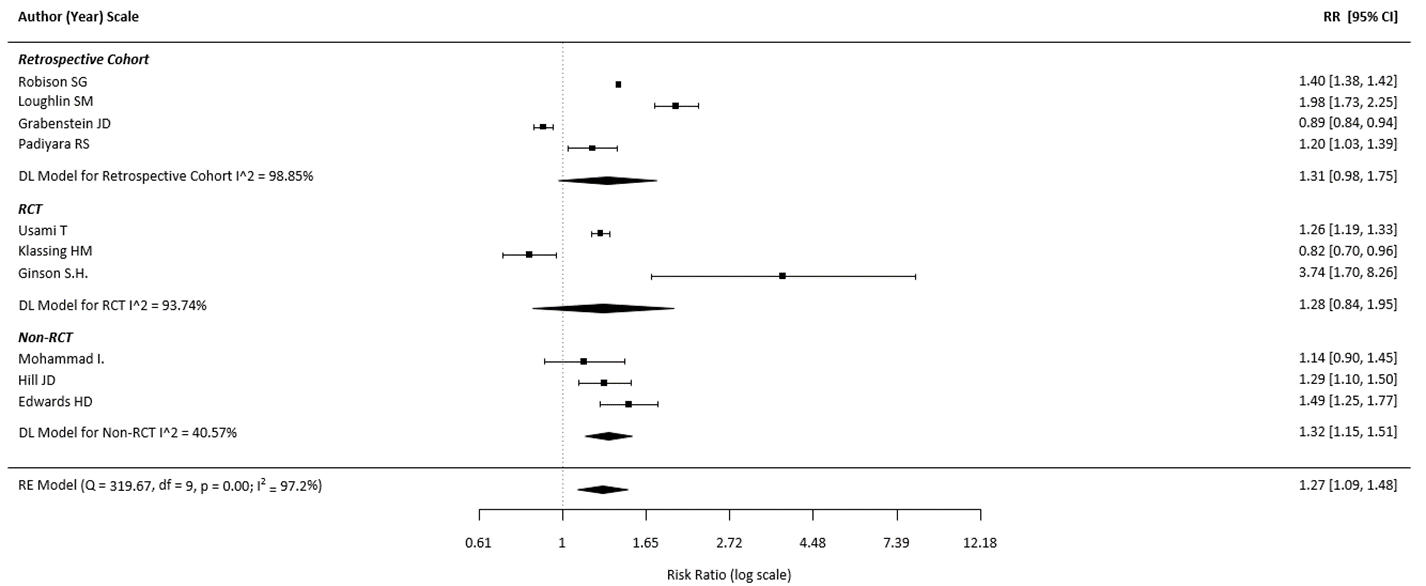


Table 3: Risk of Bias

Table 3A: Randomized Control Trials

| **First Author** | **Year** | **Random sequence generation (selection bias)** | **Allocation concealment (selection bias)** | **Blinding of participants and personnel (performance bias)** | **Blinding of outcome assessment (performance bias)** | **Incomplete outcome data (attrition bias)** | **Selective reporting (reporting bias)** | **Other sources of bias** |
| --- | --- | --- | --- | --- | --- | --- | --- | --- |
| Klassing HM | 2018 | Low | Unclear | High | High | High | Low | High |
| Usami T | 2009 | Unclear | High | High | High | Low | Low | High |
| Ginson S.H. | 2000 | Unclear | Unclear | Unclear | High | Low | Low | Unclear |

Table 3B: Observational Studies

|  |  | Selection | | | | Comparability (of cohorts on the basis on the basis of the design or analysis) | | Outcome | | | | |
| --- | --- | --- | --- | --- | --- | --- | --- | --- | --- | --- | --- | --- |
| First Author | **Year** | **Representativeness of the exposed cohort** | **Selection of the non exposed cohort** | **Ascertainment of exposure** | **Demonstration that outcome of interest was not present at start of study** | **Does the study control for the most important factor?** | **Does the study control for any additional factor(s)?** | **Assessment of outcome** | **Was follow-up long enough for outcomes to occur?** | **Adequacy of follow up of cohorts** | **Description of the lost to follow up (if available)** |  |
| Loughlin SM | 2007 | c) Selected group of users e.g. nurses or volunteers | a) Drawn from the same community as the exposed cohort | a) Secure record (e.g. surgical records) | a) Yes | a) Yes | b) No | b) Record linkage | a) Yes | a) Complete follow up - all subjects accounted for | This was a retrospective review |  |
| Padiyara RS | 2011 | c) Selected group of users e.g. nurses or volunteers | a) Drawn from the same community as the exposed cohort | a) Secure record (e.g. surgical records) | a) Yes | a) Yes | b) No | b) Record linkage | a) Yes | a) Complete follow up - all subjects accounted for | This was a retrospective review |  |
| Edwards HD | 2012 | c) Selected group of users e.g. nurses or volunteers | a) Drawn from the same community as the exposed cohort | a) Secure record (e.g. surgical records) | a) Yes | a) Yes | a) Yes | b) Record linkage | a) Yes | a) Complete follow up - all subjects accounted for | This was a retrospective review |  |
| Hill JD | 2017 | c) Selected group of users e.g. nurses or volunteers | a) Drawn from the same community as the exposed cohort | a) Secure record (e.g. surgical records) | a) Yes | b) No | b) No | b) Record linkage | a) Yes | a) Complete follow up - all subjects accounted for | This was a retrospective review |  |
| Isenor JE | 2016 | a) Truly representative of the average exposed individuals in the community | a) Drawn from the same community as the exposed cohort | a) Secure record (e.g. surgical records) | a) Yes | a) Yes | b) No | b) Record linkage | a) Yes | a) Complete follow up - all subjects accounted for | This was a retrospective review |  |
| Robison SG | 2016 | b) Somewhat representative of the average exposed individuals in the community | a) Drawn from the same community as the exposed cohort | a) Secure record (e.g. surgical records) | a) Yes | a) Yes | b) No | b) Record linkage | a) Yes | a) Complete follow up - all subjects accounted for | This was a retrospective review |  |
| Wang J | 2014 | b) Somewhat representative of the average exposed individuals in the community | a) Drawn from the same community as the exposed cohort | a) Secure record (e.g. surgical records) | a) Yes | a) Yes | a) Yes | b) Record linkage | a) Yes | a) Complete follow up - all subjects accounted for | This was a retrospective review |  |
| Grabenstein JD | 2001 | b) Somewhat representative of the average exposed individuals in the community | b) Drawn from a different source | c) Written self report | a) Yes | a) Yes | a) Yes | c) Self report | a) Yes | c) Follow up rate <90% and no description of those lost | The response rate was 51% for the Washington cohortand 55% for the Oregon cohort |  |
| Mohammad I. | 2017 | c) Selected group of users e.g. nurses or volunteers | a) Drawn from the same community as the exposed cohort | a) Secure record (e.g. surgical records) | a) Yes | a) No | a) Yes | d) No description | a) Yes | a) Complete follow up - all subjects accounted for | Same cohort pre/post intervention |  |

Table 4: Expanded Treatment Information on Intervention and Comparators

| First Author | Location | Intervention | Comparator |
| --- | --- | --- | --- |
| **Edwards HD [13]** | United States | Diabetes patients were seen by a pharmacist in a diabetes assessment service (DAS) 1 wk prior to physician apt to complete diabetes standards. EMR reviewed to ID incomplete diabetes standars of care. For DAS, pharmacist did the following: measurement of HbA1c and fasting lipid panel, comprehensive monofilament foot exam, administration of pneumococcal and influenza vaccination, collection of urine sample for screening microalbumin, referral for funduscopic eye exam, brief medication history focusing on adherence to prescribed antidiabetics/ antihypertensive and antihyperlipidemic medications and aspirin. | The control group was randomly selected from patients of 8 nonparticiping faculty physicians who were seen but had no DAS intervention |
| **Ginson SH [14]** | Canada | Patients were randomized to receive one-time pharmacist education and a standing order for influenza vaccination over 33 days. The pharmacist reviewed the benefits and potential side effects of vaccination with each patient, using a pamphlet to highlight relevant information about the vaccine. Material in the pamphlet was based on empirically derived determinants of vaccination behavior, both cognitive (fear of contracting influenza from the vaccine) and behavioral (transportation and visit time). Patients were informed that the vaccine was available in the hospital and they were asked to give written consent to be vaccinated. Eligibility and consent to be vaccinated was documented in the patient’s chart, and a conditional order for the appropriate vaccine was written by the pharmacist. The order required a physician’s signature before the vaccine could be administered. | Patients were randomized to receive standard care and admitted to the Family Practice Program by physician over 33 days. |
| **Grabenstein JD [15]** | United States | Patients received the influenza vaccination in Washington state. Washington state regulations explicitly authorize pharmacists to administer medications. | Patients received the influenza vaccination in Oregon state. Oregon regulations did not explicitly authorize drug administration by pharmacists in 1997 and 1998, nor were any pharmacists known to do so. |
| **Hill JD [16]** | United States | Ptients received standard care and the pharmacy technician intervention during the last 2 weekends over a fourconsecutive weekend study period in October 2011. Targeted pharmacy technician interventions consisted of phone call reminders and/or face-to-face discussions with nursing staff. It also consisted of a review of the immunization status of all patients on the Cardiovascular Progressive Care unit at the University of Kansas HealthSystem and the generation of a list of patients who had not been screened or immunized. Pharmacy technicians notified nurses by phone or direct contact, of patients on the unit who required follow-up to complete the appropriate vaccination documentation. Nursing retained responsibility during the intervention period for completing follow-up by utilizing the immunization screening questions outlined in the vaccine order set and administering or documenting a reason for lack of administration of the appropriate vaccination. Pharmacy technicians received standardized training which reviewed generating the patient list and identifying patients which needed additional follow-up within the electronic medical record (EMR). A script was developed for pharmacy technicians to utilize when speaking with nursing staff which also provided a standard list of expected questions and suggested answers. Pharmacy technicians were advised to complete their first follow-up discussion with nursing by 10 am each morning of the study period. | Patients received standard care during the first 2 weekends over a fourconsecutive weekend study period in October 2011. Standard of care treatment consisted of nursing ownership, through a standing order program (SOP), of the immunization administration and documentation process |
| **Klassing HM [18]** | United States | Patients were randomized to receive a phone call intervention. A phone call script was utilized for the phone call intervention; patient specific questions were fielded on an individual basis. The phone call script referenced 2014 Center for Disease Control and Prevention (CDC) immunization schedule and guidelines. All subjects were exposed to in-store advertising for the seasonal influenza vaccine and received flyers advertising on-site immunizations when picking up prescriptions during the study period. A brief voicemail with a direct contact phone number was left for phone calls that were unanswered. | Patients were randomized to receive no vaccination information. All subjects were exposed to in-store advertising for the seasonal influenza vaccine and received flyers advertising on-site immunizations when picking up prescriptions during the study period. |
| **Loughlin SM [19]** | United States | Patients participated in a pharmacist-managed influenza vaccination program during the 2004-2005 influenza season. During the 2004–2005 influenza season, the clinical pharmacist, residents, and students certified in immunization delivery screened patients and offered the influenza vaccination, under a standing-order protocol, to all patients treated at the lipid clinic as part of their usual activities. | Patients did not participate in a formal immunization program at Kelsey-Seybold during the 2003-2004 influenza season. Clinics independently screened patients for influenza vaccination. |
| **Mohammad I [20]** | United States | Patients were enrolled in pharmacist-led chronic care mangement. Chronic care management focuses on medication management, care coordination, and management at transitions of care. | Patients received usual care treatment. |
| **Padiyara RS [21]** | United States | Patients received education, directly managed drug therapy and preventive care services for 12 months all provided by a pharmacist. Pharmacists had autonomy in assessing patients, providing disease-state education, reviewing current medication lists, initiating or adjusting medication therapy, ordering laboratory tests, and determining appropriate follow-up. Pharmacists met with new patients for approximately 45 minutes at the initial visit and returning patients for approximately 30 minutes per visit. | Patients received usual care for 12 months. Standard care includes the provision of preventive care services and screenings; medication management; education of the patient by the primary care physician (PCP), nurse practitioners or physician assistants working under the supervision of the PCP, or nursing staff working directly with the PCP; and referral to other physician specialists (e.g., endocrinology) if deemed appropriate by the PCP. |
| **Robison SG [22]** | United States | Participants were allowed to be immunized by a pharmacist without a prescription after the Oregon law change in 2011. Participants were observed from 2011 to 2014. Under the rule changes, an adolescent pharmacy immunization protocol was jointly developed and administered by the Oregon Board of Pharmacy and the Oregon Public Health Division. | Participants were allowed to be imunized by a pharmacist in Oregon by prescription only before 2011. Participants were observed from 2007 to 2010. |
| **Usami T [23]** | Japan | Pharmacists in the intervention pharmacy group displayed two posters, provided participants with information on the risks of influenza and benefits of the vaccine in addition to the information in a leaflet and on the two posters, and physically placed the leaflet in the hands of the participants. The leaflet and two posters contained information on influenza susceptibility and severity, vaccine efficacy, cost, and sites where vaccinations were available to elderly residents. | Patients in the control pharmacy group were not shown a pharmacist poster or an information leaflet containing information on influenza susceptibility and severity, vaccine efficacy, cost, and sites where vaccinations were available to elderly residents. |
